# Outbreaks of *Serratia marcescens* and *Serratia rubidaea* bacteremia in a central Kathmandu hospital following the 2015 earthquakes

**DOI:** 10.1093/trstmh/try077

**Published:** 2018-08-11

**Authors:** Abhilasha Karkey, Niva Joshi, Shiva Chalise, Suchita Joshi, Shrijana Shrestha, To Nguyen Thi Nguyen, Sabina Dongol, Buddha Basnyat, Stephen Baker, Christine J Boinett

**Affiliations:** 1 Oxford University Clinical Research Unit, Patan Academy of Health Sciences, Satdobato, Lagankhel Rd, Patan, Kathmandu, Nepal; 2 Hospital for Tropical Diseases, Wellcome Trust Major Overseas Programme, Oxford University Clinical Research Unit, 764 Vo Van Kiet Street, Quan 5, Ho Chi Minh City, Vietnam; 3 Centre for Tropical Medicine and Global Health, Nuffield Department of Medicine and Research Building, Oxford University, Old Campus Road, Roosevelt Drive, Headington, Oxford, UK; 4 Patan Academy of Health Sciences, Department of Paediatrics, Patan Hospital, Satdobato Lagankhel Rd, Patan, Kathmandu, Nepal; 5 Department of Medicine, Addenbrookes Hospital, Box 157, Hills Road, Cambridge, UK

**Keywords:** bloodstream infections, natural disaster infection outbreak, *Serratia marcescens*, *Serratia rubidaea*, *Serratia* spp

## Abstract

**Background:**

Human infections with *Serratia* spp. are generally limited to *Serratia marcescens* and the *Serratia liquefaciens* complex. There is little data regarding the infections caused by the remaining *Serratia* spp., as they are seldom isolated from clinical specimens.

**Methods:**

In this health care setting in Kathmandu, Nepal routine blood culture is performed on all febrile patients with a temperature >38°C or when there is clinical suspicion of bacteremia. During 2015 we atypically isolated and identified several *Serratia* spp. We extracted clinical data from these cases and performed whole genome sequencing on all isolates using a MiSeq system (Ilumina, San Diego, CA, USA).

**Results:**

Between June and November 2015, we identified eight patients with suspected bacteremia that produced a positive blood culture for *Serratia* spp., six *Serratia rubidaea* and five *Serratia marcescens*. The *S. rubidaea* were isolated from three neonates and were concentrated in the neonatal intensive care unit between June and July 2015. All patients were severely ill and one patient died. Whole genome sequencing confirmed that six Nepalese *S. rubidaea* sequences were identical and indicative of a single-source outbreak.

**Conclusions:**

Despite extensive screening we were unable to identify the source of the outbreak, but the inferred timeline suggested that these atypical infections were associated with the aftermath of two massive earthquakes. We speculate that deficits in hygienic behavior, combined with a lack of standard infection control, in the post-earthquake emergency situation contributed to these unusual *Serratia* spp. outbreaks.

## Introduction

The Gram-negative bacterial genus *Serratia* is found within the broad Enterobacteriaceae family and is currently differentiated into 10 species.^1^ Human infections with *Serratia* are not as common as with more virulent members of the Enterobacteriaceae (e.g., *Salmonella, Klebsiella, Escherichia coli*), but when they do arise they are largely associated with *Serratia marcescens* and the *Serratia liquefaciens* complex, which includes *S. liquefaciens*, *Serratia proteomaculans* and *Serratia grimesii.*^1,2^ There is little data regarding the infections caused by the remaining *Serratia* species, as they are seldom isolated from clinical specimens.^2^

*S. marcescens* is the primary species within the *Serratia* genus associated with disease. The organism can be isolated from various clinical specimens (e.g., blood, tracheal aspirates, urine) when associated with infection, and health care–related outbreaks of *S. marcescens* are well described.^3–5^*S. marcescens* thrives in moist environments, which can include intravenous solutions, indwelling intravenous catheters, soaps and disinfectants, all of which have been described as the source of outbreaks.^4–7^ Notably, although some cases have been reported, *S. marcescens* rarely causes invasive community-acquired infections among nonimmunocompromised individuals.^8,9^

*Serratia rubidaea* is a less well-described member of the genus and is chiefly found in soil, water and food. The isolation of this organism from clinical specimens is rare,^10,11^ but it can cause opportunistic infections in severely ill patients receiving broad-spectrum antimicrobials or those that have undergone surgery or other invasive procedures.^3,10,12–14^ When identified in clinical specimens, *S. rubidaea* is largely isolated from respiratory tract samples, skin wounds, feces and bile.

## Materials and methods

Patan Hospital is a 450-bed general hospital that serves the local population in the Lalitpur area of the central Kathmandu valley. All neonates in which the *Serratia* infections arose were among those neonates who were born in Patan Hospital; all other patients with *Serratia* infections had presented with fever to the emergency department of Patan Hospital. All procedures were reviewed and approved by the institutional review board (OxTREC 24-16) as well as the Nepal Health Research Council (278/2015).

Blood cultures are performed routinely on all febrile patients with a temperature >38°C or when there is clinical suspicion of bacteremia. For blood cultures in pediatric patients, 3–5 mL of venous blood was drawn and inoculated into BACTEC Peds Plus aerobic bottles following the manufacturer’s recommendations (Becton Dickinson, Franklin Lakes, NJ, USA). All inoculated bottles were incubated at 37°C in a BACTEC 9050 analyzer for 7 days^15^ or until flagged growth in the automated system. Organisms were identified by standard methods, including API20E identification kits (Bio-Mérieux, Craponne, France). Antimicrobial susceptibility testing was performed at the time of isolation by the modified Kirby–Bauer disc diffusion method. Zone size interpretations were performed following the Clinical and Laboratory Standards Institute guidelines. The organisms were tested against 15 antimicrobials (Table 1).

**Table 1.** Summary of *Serratia* spp. isolates from blood cultures

ID	Date	Sequencing ID	Acc. no.^c^	Ward^d^	AMX	CTX	CIP	SXT	GEN	AMK	OFX	CHL	MEM	SAM	CST	TZP	IMP	TGC	Outcome
2167^a^	16/06/15	*S. rubidaea*	ERS1978223	NICU	R	I	S	R	S	S	S	R	S	R	R	S	S	S	Survived
2196^a^	06/07/15	*S. rubidaea*	ERS1978224	NICU	R	I	S	R	S	S	S	R	S	R	R	S	S	S	
2188^b^	27/06/15	*S. rubidaea*	ERS1978225	NICU	R	S	S	S	S	S	S	R	S	R	R	S	S	S	Survived
2195^b^	06/07/15	*S. rubidaea*	ERS1978226	NICU	R	S	S	S	S	S	S	R	S	R	R	S	S	S	
2208^b^	19/07/15	*S. rubidaea*	ERS1978227	NICU	R	S	S	S	S	S	S	R	S	R	S	S	S	S	
2199	07/07/15	*S. rubidaea*	ERS1978228	NICU	R	R	S	I	S	S	S	R	S	R	R	R	S	S	Died
2280	28/09/15	*S. marcescens*	ERS1978229	ER	R	S	S	S	S	S	S	R	S	S	R	S	S	S	Died
2301	18/10/15	*S. marcescens*	ERS1978230	PW	R	S	S	I	I	S	S	R	S	S	R	S	I	R	Survived
2313	10/11/15	*S. marcescens*	ERS1978231	NICU	R	S	S	S	S	S	S	R	S	S	R	S	S	S	Survived
2314	10/11/15	*S. marcescens*	ERS1978232	PW	R	S	S	R	S	S	S	R	S	S	R	S	S	S	Survived
2315	10/11/15	*S. marcescens*	ERS1978233	PW	R	S	S	S	S	S	S	R	S	S	R	S	S	S	Survived

^a^^,b^Same patient with repeat positive blood cultures.

^c^Accession numbers for the individual *Serratia* isolates in this study that were submitted to the European Nucleotide Archive.

^d^NICU: Neonatal Intensive Care Unit; ER: Emergency Room; PW: Pediatric Ward.

AMX: amoxicillin; CTX: cefotaxime; CIP: ciprofloxacin; SXT: cotrimoxazole; GEN: gentamicin; AMK: amikacin; OFX: ofloxacin; CHL: chloramphenicol; MEM: meropenem; SAM: ampicillin-sulbactam; CST: colistin; TZP: piperacillin-tazobactam; IMP: imipenem; TGC: tigecycline.

Genomic DNA was extracted using the Wizard genomic DNA purification kit (Promega, Madison, WI, USA) and sequenced on a MiSeq system (Ilumina, San Diego, CA, USA). A total of 300 base paired end reads were subjected to *de novo* assembly using Velvet^16^ and annotated using Prokka.^17^ The resulting pan-genome, consisting of 1945 genes, was generated using Roary^18^ with the 11 novel genome sequences from this study and 11 *Serratia* spp. sequences accessed from the National Center for Biotechnology Information. Single-nucleotide polymorphism (SNP) sites were extracted from a multi-FASTA alignment of the core genomes of the 22 sequences^18^ and a maximum likelihood phylogenetic tree was generated using RAxML using a Generalized Time Reversal model with a gamma correction and 100 bootstrap replicates.^19^ Antimicrobial resistance genes were identified using ARIBA software^20^ using the Comprehensive Antibiotic Resistance Database.^21^ Results were viewed in Phandango.^22^

Environmental sampling for the neonatal intensive care unit (NICU) was performed after the identification of the first *Serratia* spp. in the blood. Swab samples were taken from the air conditioner filters and linings, window linings, sink surfaces, medicine trolley, weighing machine, door handles and phone sets. All swabs were plated on MacConkey and blood agar and incubated for 48 h at 37°C. Bacterial isolates were identified by standard methods, including API20E identification kits (Bio-Mérieux).

## Results

Between June and November 2015, 11 *Serratia* spp. (6 *S. rubidaea* and 5 *S. marcescens*) were isolated from blood cultures in this health care facility (Table 1). In comparison, *Serratia* spp. had not been isolated in this hospital between 1992 and 2016. The six *S. rubidaea* samples (2167, 2196, 2188, 2195, 2208 and 2199) were isolated in June and July in the NICU from three children (some repeat samples) born at the hospital in the preceding days. The five *S. marcescens* samples (2301, 2313, 2314, 2315 and 2280) were isolated from five patients from either the pediatric ward, the NICU or the emergency department between September and November (Table 1). All five patients with *S. marcescens* bacteremia presented to the hospital with high-grade fever (>39°C) of >3 d. The blood samples taken on the day of hospital admission (day 1) produced a positive culture for *S. marcescens*, suggesting that all infections were community acquired. Available medical records did not identify any notable risk factors for infection and reported the death of one of the patients (Table 1).

Given the rarity and the temporal clustering of the *S. rubidaea* cases, we extracted the hospital notes from the three patients with these infections. Patient 1 (2167) was associated with a female born via a vaginal breech delivery at 29 weeks of gestation. The mother had had no complications during pregnancy, but the child had a low birth weight (1.1 kg), with APGAR scores of 2/10 and 4/10 at 1 and 5 min after birth, respectively. Resuscitation was performed and she was intubated. The child was transferred immediately to the NICU, where she was given respiratory support through continuous positive airway pressure (CPAP). On admission to the NICU her heart rate was 165 bpm and her blood pressure was 55/28 mmHg. A blood culture was requested on day 1 in the NICU and she was empirically administered ampicillin and amikacin for 5 d. The results of the primary blood culture were negative, but due to deterioration in her condition and the development of fever on day 5 she was administered meropenem and colistin. There was no clinical improvement and a blood culture was repeated on day 17, which resulted in the isolation of *S. rubidaea*. Antimicrobials meropenem and colistin were continued, but due to the lack of clinical improvement, a repeat blood culture was performed on days 20, 24 and 26, all of which were negative. As the fever had not subsided, the child was switched to ofloxacin and amikacin. The fever still did not subside and on day 37 a blood culture was again performed, resulting in the isolation of *S. rubidaea* that was resistant to amoxicillin, chloramphenicol, ampicillin-sulbactam and colistin but susceptible to other available antimicrobials (Table 1). The neonate was placed on a combination of vancomycin and meropenem. The fever began to subside on day 41 and on day 44, after a sterile blood culture, the infant was transferred to the nursery. The infant remained in the nursery until day 92 of life, when she had a body weight of 1.48 kg and was discharged.

After this primary case of *S. rubidaea* bacteremia we conducted rigorous environmental sampling of the NICU, however, the culture results were inconclusive. As the procedure of fumigation can reach all spaces and eliminate a host of bacteria with long-lasting effects, the hospital, according to available infection control guidelines, decided to perform the procedure in the NICU. Formaldehyde fumigation of the NICU was performed on 19 June, but patient 2 (2188) was identified on 29 June—a female newborn delivered after an emergency cesarean at 36 weeks. The mother was hospitalized with preeclampsia and intrauterine growth retardation during the third trimester. The neonate was of low birth weight (1 kg), was in respiratory distress (respiratory rate 36) and admitted to the NICU, where she was given respiratory support with CPAP. The child was treated empirically with ampicillin and amikacin for 5 d. On the third day after delivery, an endocervical swab of the mother returned growth of *Klebsiella pneumoniae* that was resistant to amoxicillin, cefotaxime and cotrimoxazole but susceptible to ciprofloxacin and chloramphenicol. The neonate developed fever on day 9 and was prescribed meropenem, colistin, aminophylline and fluconazole. There was no improvement in the condition of the child and on day 17 in the NICU, a blood culture (2195) was positive for *S. rubidaea* that was resistant to amoxicillin, chloramphenicol, ampicillin-sulbactam and colistin but susceptible to other available antimicrobials (Table 1). The neonate had intermittent CPAP support and on day 19 she was placed on a ventilator. An additional blood culture (2208), performed on day 26 in the NICU, remained positive for *S. rubidaea* and had an identical antimicrobial susceptibility pattern as the isolate recovered from the blood culture on day 17. Treatment was switched to cefotaxime and ofloxacin for 5 d. The infant remained febrile and a blood culture performed on day 33 returned *S. rubidaea* that exhibited resistance to cefotaxime with the same susceptibility profile as the primary bloodstream isolate. The neonate was switched to meropenem, amikacin and ofloxacin. The fever began to subside on day 34, but *S. rubidaea* was again isolated from a blood culture on day 37. Despite the continued isolation of *S. rubidaea* from blood, the neonate improved and, once afebrile (day 40), she was transferred to the nursery where she began to gain weight. On reaching a body weight of 1.8 kg (day 75 of life) she was discharged.

A third patient (2199) on 7 July suggested either inadequate fumigation of the NICU or human-to-human contact transmission. The neonate was born through assisted vaginal delivery at 28 weeks of gestation. An sonogram performed 1 month prior to delivery suggested a normal pregnancy with a fundal posterior placenta. The mother had a history of primary infertility and had conceived after a dilation and curettage surgery. The mother also had pelvic discharge during the third trimester and a urine culture resulted in the isolation of *K. pneumoniae* that was resistant to ampicillin, cefotaxime, cotrimoxazole, nitrofurantoin and nalidixic acid. The premature neonate was a male of low birth weight (1.1 kg), with an APGAR score of 7/10 and 8/10 at 1 and 5 min after birth, respectively. After birth, the neonate developed secondary apnea; bagging was performed for two cycles and then he was intubated and transferred to the NICU. He was administered ampicillin, amikacin and cefotaxime after a blood sample had been collected for microbiological culture. On day 2, the infant went into septic shock, with a heart rate of 172 bpm and a blood pressure of 44/15 mmHg. A blood culture performed on day 1 was positive for *K. pneumoniae* and the isolate exhibited an identical antimicrobial susceptibility pattern as the organism previously isolated from the mother. The infant was administered dopamine and a fluid bolus on two occasions and was treated with colistin and meropenem. The neonate remained febrile and the umbilical venous catheter (UVC) tip along with a repeat blood sample was sent for microbiological culture on day 14. Both cultures were positive for *S. rubidaea* that was resistant to cefotaxime, tigecycline, chloramphenicol and cotrimoxazole but was susceptible to other available antimicrobials (Table 1). The neonate remained febrile and was unable to gain weight. On day 27, due to his prematurity and deranged coagulation secondary to septicemia, he developed a grade IV intraventricular hemorrhage with periventricular leukomalacia and bilateral hydrocephalus. It was decided that ventilator support would be withdrawn and the child subsequently died.

Extensive infection control precautions were taken. Rigorous hand hygiene was observed whereby hands and equipment were cleaned and disinfected on the way into the patient’s room and on the way out again. Environmental hygiene was observed through continuous cleaning and disinfection. The mothers of the neonates were included in the infection prevention protocols, which helped in the maintenance of a clean and sanitary environment. Neonates who came for admission into the NICU were screened and all microbiological data were surveyed to ensure that there were no further outbreaks. Following these precautions, no further cases of *Serratia* spp. were reported.

**Figure 1.**
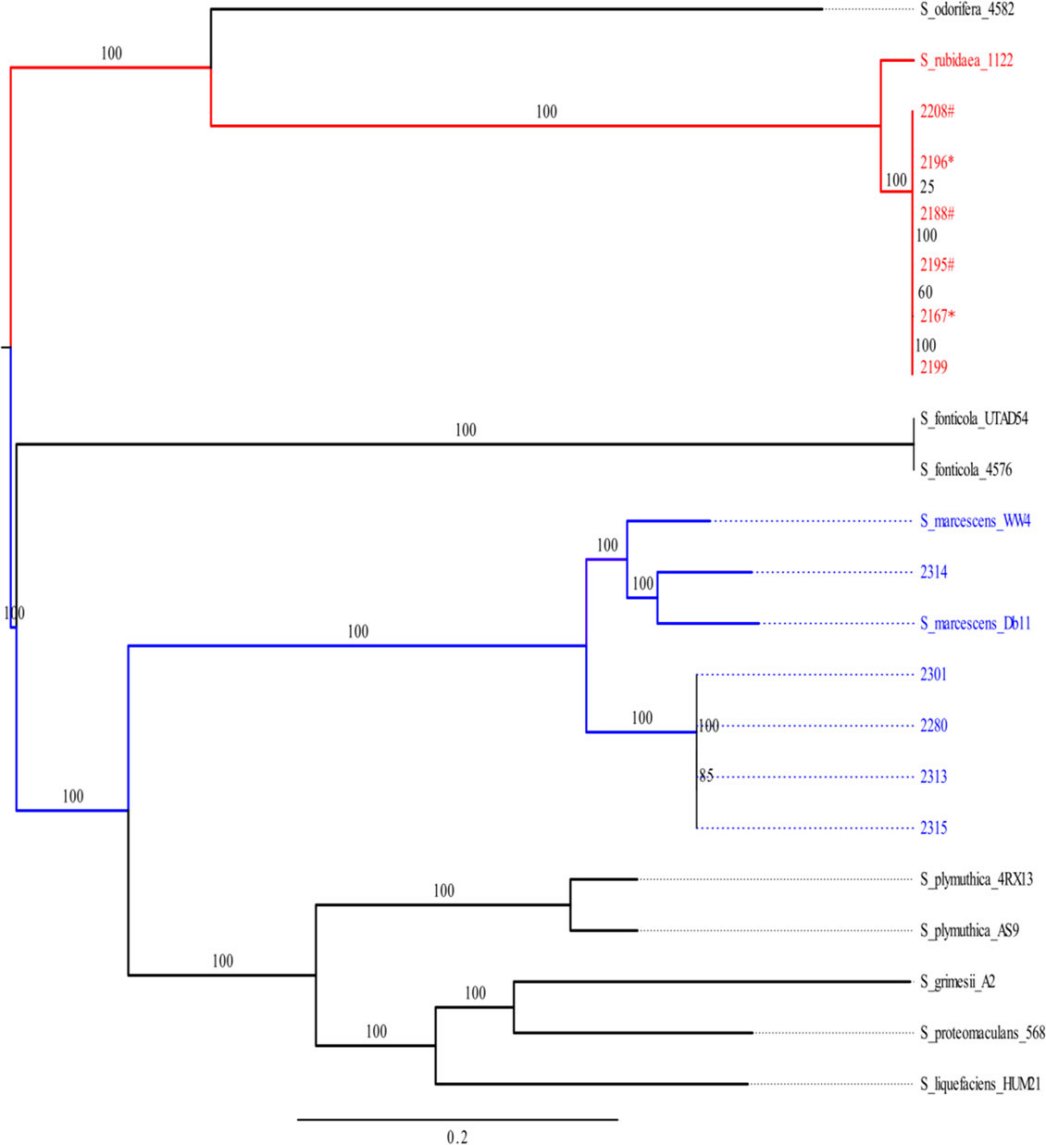
The phylogenetic relationship of *Serratia* associated with bacteremia in Nepal. Maximum likelihood phylogenetic tree generated using RAxML of the 11 novel Nepalese *Serratia* sequences and the reference genomes of *S. odorifera*_4582 (DBY01000000), *S. rubidaea*_112 (CP014474), *S. fonticola*_UTAD54 (AUZV01000000), *S. fonticola*_4576 (CP011254.1), *S. marcescens* WW4 (CP003959), *S. marcescens* Db11 (HG326223), *S. plymuthica*_4RX13 (CP006250), *S. plymuthica*_AS9 (CP002773), *S. grimesii*_A2 (JGVP00000000), *S. proteomaculans*_568 (CP000826) and *S. liquefaciens* (CP011303). Branches are numbered with bootstrap values and the scale corresponds to the number of substitutions per site.

To add further context to the isolation of these organisms, the six *S. rubidaea* and five *S. marcescens* samples were subjected to whole genome sequencing. The phylogenetic structure of 22 *Serratia* sequences (including reference sequences) confirmed the subspecies classification generated using conventional biochemical testing, segregating the *S. rubidaea* and *S. marcescens* into distinct lineages (Figure 1). The six Nepalese *S. rubidaea* sequences were identical (indicative of a single-source outbreak) and clustered alongside the sequence of *S. rubidaea* isolated from a patient in China,^14^ varying by 298 SNPs from this sequence. The *S. marcescens* sequences clustered alongside the previously sequenced Db11^23^ and WW4^24^ organisms. In a comparable fashion to the *S. rubidaea*, we observed that four of the *S. marcescens* organisms were all identical, and again likely part of an isolated outbreak in the community. Notably, *S. marcescens* 2314 was distantly related to the other organisms, sitting adjacent to the two reference genomes. All 11 of the *Serratia* isolates were phenotypically resistant to amoxicillin, ampicillin-sulbactam, chloramphenicol and colistin. However, genotypic analysis found only an *aac(6*′*)Ic* gene, which was located on the chromosome and confers resistance to aminoglycosides; isolates were found to be susceptible to gentamicin (Table 1). This lack of genotype support for the antimicrobial susceptibility was not surprising, as *Serratia* spp. are intrinsically resistant to multiple antimicrobials, including colistin and some broad-spectrum β-lactams, and the resistance gene content may not be associated with *in vitro* phenotypic observations.^2,25^

## Discussion

All these cases of *Serratia* spp. presented within a few weeks of two massive earthquakes, which occurred on 25 April (7.8 Richter) and 12 May (7.3 Richter), with continuous aftershocks reaching magnitudes of up to 6.7 Richter. It is known that natural disasters, including earthquakes, often lead to an increase in specific infectious diseases.^26^ The physical structure of the hospital was severely damaged during these earthquakes and various hospital facilities, including surgery and obstetric birthing centers, were temporarily relocated to tents outside of the hospital for up to 4 months after the earthquake. All the neonates with *S. rubidaea* bacteremia had been born in these tents and were likely exposed to a higher infection risk in the postearthquake disorder. All of the cases were low birth weight, premature neonates, which are generally predisposed to acquiring infections. Considering the time span of the *S. rubidaea* infections and their genetic relatedness, we surmise that these cases were an atypical outbreak restricted to the NICU. The first neonate was likely infected through a reservoir in the soil or through the mother during delivery in the tents, with then successive cases being infected within the NICU during treatment procedures such as intubation or catheterization or simply through hand transmission from the health care providers. The pathogen will often lodge first in the gastrointestinal tract and will then translocate to other sites such as the urinary or respiratory tracts. From there the organisms were likely picked up in the blood following constant seeding. Generally in a NICU the source of an outbreak of infection with Gram-negative bacilli is a fairly constant one, such as the water supply, but it seems from the evidence here that the mothers were a more likely the source of the *S. rubidaea* and that the labor wards were most likely involved.

Comparatively, the *S. marcescens* cases presented to the hospital with presentations indicative of community-acquired infections, which is again atypical for cases of *S. marcescens*. Comparable to the *S*. *rubidaea* isolates, the majority of the *S. marcescens* were genetically identical. These data therefore suggest that these cases were an isolated community outbreak. We were unable to confirm an epidemiological association between cases due to the lack of post-earthquake follow-up data.

Our observations outline atypical epidemiological observations of *Serratia* spp. infections in a hospital setting in the post–2015 earthquake period in Kathmandu. The earthquakes resulted in substantial disruption of medical care and interventions, which limited our outbreak investigation, as we were unable to carry out environmental sampling. Additionally, infection control and surveillance for nosocomial infections are not well established in Nepal. Rapid hygienic interventions in the hospital and preemptive screening of patients are known to be effective in overcoming hospital outbreaks. We consider that the deficits in hygienic behavior combined with the lack of standard infection control measures during emergency conditions likely contributed to this outbreak.
